# Laser Ablation Facilitates Implantation of Dynamic Self-Regenerating Cartilage for Articular Cartilage Regeneration

**DOI:** 10.3390/jfb15060148

**Published:** 2024-05-29

**Authors:** Yingfang Fan, Fernando P. S. Guastaldi, Gem Runyan, Ying Wang, William A. Farinelli, Mark A. Randolph, Robert W. Redmond

**Affiliations:** 1 Wellman Center for Photomedicine, Massachusetts General Hospital, Harvard Medical School, Boston, MA 02144, USA; yfan8@mgh.harvard.edu (Y.F.); grunyan@luc.edu (G.R.); ywang29@mgh.harvard.edu (Y.W.); bfarinelli@mgb.org (W.A.F.); 2 Plastic Surgery Research Laboratory, Department of Surgery, Massachusetts General Hospital, Harvard Medical School, Boston, MA 02114, USA; marandolph@mgh.harvard.edu; 3 Division of Oral and Maxillofacial Surgery, Department of Surgery, Massachusetts General Hospital, Harvard School of Dental Medicine, Boston, MA 02114, USA; fguastaldi@mgh.harvard.edu

**Keywords:** cartilage, fractional laser, implant, cartilage integration, cartilage regeneration, neocartilage

## Abstract

Objectives: This study investigated a novel strategy for improving regenerative cartilage outcomes. It combines fractional laser treatment with the implantation of neocartilage generated from autologous dynamic Self-Regenerating Cartilage (dSRC). Methods: dSRC was generated in vitro from harvested autologous swine chondrocytes. Culture was performed for 2, 4, 8, 10, and 12 weeks to study matrix maturation. Matrix formation and implant integration were also studied in vitro in swine cartilage discs using dSRC or cultured chondrocytes injected into CO_2_ laser-ablated or mechanically punched holes. Cartilage discs were cultured for up to 8 weeks, harvested, and evaluated histologically and immunohistochemically. Results: The dSRC matrix was injectable by week 2, and matrices grew larger and more solid with time, generating a contiguous neocartilage matrix by week 8. Hypercellular density in dSRC at week 2 decreased over time and approached that of native cartilage by week 8. All dSRC groups exhibited high glycosaminoglycan (GAG) production, and immunohistochemical staining confirmed that the matrix was typical of normal hyaline cartilage, being rich in collagen type II. After 8 weeks in cartilage lesions in vitro, dSRC constructs generated a contiguous cartilage matrix, while isolated cultured chondrocytes exhibited only a sparse pericellular matrix. dSRC-treated lesions exhibited high GAG production compared to those treated with isolated chondrocytes. Conclusions: Isolated dSRC exhibits hyaline cartilage formation, matures over time, and generates contiguous articular cartilage matrix in fractional laser-created microenvironments in vitro, being well integrated with native cartilage.

## 1. Introduction

Joint function depends on smooth articulating surfaces provided by healthy articular cartilage at the joint interface [1]. Trauma or disease can adversely modify cartilage surfaces, leading to the loss of cartilage and an irregular cartilage surface that impacts joint function [2]. Focal cartilage defects may result from trauma, overuse, recreational activity, or excessive load bearing or be secondary to other injuries [3]. Inflammation and progression to osteoarthritis further complicate joint performance [4].

Cartilage has little native regenerative ability once injury or degradation has occurred [5,6,7]. Current clinical treatments focus on implanting healthy cartilage from the non-weight-bearing regions of the joint (osteoarticular transfer system (OATS) or mosaicplasty) or invasive microfracture to promote the influx of bone marrow cells (BMCs) into the lesion [8,9]. Although OATS restores the articular surface with plugs of hyaline cartilage, there is limited integration of the plugs with the adjacent plugs and the surrounding native tissue, which may limit the durability of the repair. Microfracture, on the other hand, permits the invasion of putative chondrogenic stem cells into the defect to promote repair, but the new tissue formed often contains more type I collagen found in typical scar tissue and lacks the predominant type II collagen found in normal hyaline cartilage. Thus, a pressing unmet need remains for a new clinical approach to true hyaline articular cartilage regeneration and resurfacing.

There has been great research interest in tissue engineering approaches for repairing cartilage injury or disease. To generate presumptive new hyaline cartilage, autologous cells (mesenchymal stem cells or chondrocytes) are typically implanted directly (autologous chondrocyte implantation, ACI) or as a cell-seeded matrix (matrix-induced autologous chondrocyte implantation, MACI) into a cartilage defect [10,11,12,13,14,15]. Although these approaches generate new tissue in the defect, the question remains whether the neotissue formed using these methods is any improvement over microfracture [16]. Furthermore, the biomechanical performance of the neotissue is typically poor [17,18,19,20]. New approaches are required to address these limitations, allowing for effective cartilage regeneration and improved joint performance.

Most tissue engineering approaches utilize primary chondrocytes or stem cells seeded into three-dimensional porous scaffolds or gels to stimulate new extracellular matrix (ECM) formation [21,22]. However, under the correct culture conditions, chondrocytes have an innate capacity for generating limited pericellular ECM without exogenous scaffold material [23,24]. Meppelink et al. advanced this concept by placing chondrocytes suspended in hypoxic media in oscillatory motion to form a dynamic Self-Regenerating Cartilage (dSRC) matrix [25]. These conditions promote chondrocyte aggregation and ECM production with characteristics similar to native hyaline cartilage. Exploiting this method for scaffold-free cartilage matrix formation could be part of a combinatorial approach to cartilage repair and regeneration.

Symptomatic cartilage defects on the centimeter scale do not regenerate well and tend to generate fibrocartilage comprised of collagen type I rather than II-rich hyaline cartilage [26]. In our prior study, a mechanical punch was used to create lesions. These were inevitably of an osteochondral nature as it is virtually impossible to limit the lesion depth to the cartilage layer alone. As a result, blood can seep into the lesion. Not only does this interfere with implant placement but it may also have additional confounding factors due to the possible influence of bone marrow stem cells in regeneration (as in microfracture). In contrast, laser ablation provides a means for the exquisite control of lesion depth, with the ability to confine lesions within the cartilage layer itself, removing any influence of blood in the regenerative process and allowing us to focus solely on the influence of the SRC implant in situ. The mechanistic study reported here allowed us to test whether the laser ablation mechanism would have any detrimental effects on the host tissue (e.g., thermal damage to tissue and cytotoxicity) or negatively affect the host/implant interface and impact the integration of neotissue with the host. We hypothesized that creating lesions of this type in cartilage would result in functional regeneration rather than dysfunctional scarring, paving the way for a potential clinical approach to cartilage rejuvenation. In contrast to other methods that are limited by lack of integration of the implant with the host (OATS), fibrocartilage formation (ACI, microfracture), or the disruption of the osteochondral junction, the laser ablation/dSRC approach can actually generate a true hyaline neocartilage implant, intimately integrated into the host tissue at the purely chondral level. Additionally, the ablative process has the ability to pre-sculpt a lesion shape to better receive and retain the dSRC implant for the regeneration and integration of neocartilage.

## 2. Methods

### 2.1. Chondrocyte Harvest

Articular cartilage was harvested in a sterile fashion from the distal femur in the knees of 3- to 4-month-old swine that were euthanized in other studies. The cartilage was dissected, rinsed in PBS (Sigma, St. Louis, MO, USA), chopped into ~1-mm^3^ pieces, and then digested in Ham’s F-12 medium (Invitrogen, Carlsbad, CA, USA), containing 0.1% collagenase type 2 (Worthington Biochemical, Freehold, NJ, USA) and 1% antibiotic/antimycotic solution for 14–18 h at 37 °C in a Heratherm IMH180 oven (Thermo Electron LED GmbH, Langenselbold, Germany). Undigested debris was removed by passing the cell suspension through a 100 µm filter (Falcon, Corning, NY, USA). Extracted chondrocytes were subsequently washed three times with a “chondrocyte medium” (see below). Cell viability was assessed using a trypan blue (Sigma, St. Louis, MO, USA) exclusion assay. Only chondrocyte harvests exhibiting a viability of ≥90% were used. Cell counts were performed using a hemocytometer, and cell suspensions were adjusted to 2 × 10^6^ cells/mL.

### 2.2. Chondrocyte Culture

Chondrocytes were cultured in a “chondrocyte medium” of Ham’s F-12 medium supplemented with 10% fetal bovine serum (Invitrogen), 1% antibiotic/antimycotic solution, 1% non-essential amino acids (Invitrogen), and 50 mg/mL of ascorbic acid [26].

### 2.3. Formation and Maturation of dSRC

To form the dSRC, 10^7^ freshly harvested autologous swine chondrocytes were placed in 5 mL of chondrocyte medium in 15 mL polypropylene tubes, sealed, and cultured horizontally in a rack, under an oscillating motion at 40 cycles per minute for 14 days at 37 °C in a Heratherm IMH180 oven (Thermo Electron). During this time, the chondrocytes aggregated and began to generate a new extracellular matrix to form a pellet or sheet of dSRC. The in vitro maturation of dSRC was investigated after the culture for up to 12 weeks. Media changes were performed every 3–4 days.

### 2.4. Cell Count

The total cell number in dSRC pellets was calculated after up to 14 weeks of culture in vitro. Ten samples from each group were analyzed. Briefly, dSRC was collected and digested with Ham’s F-12 medium (Invitrogen, Carlsbad, CA, USA), containing 0.1% collagenase type 2 (Worthington Biochemical, Freehold, NJ, USA) and 1% antibiotic/antimycotic solution at 37 °C for 6 h. After digestion, cell debris and insoluble materials were removed via centrifugation at 6000× *g* for 5 min. Portions of the digest were analyzed for total cell count using a hemocytometer.

### 2.5. Fractional Treatment of Ex Vivo Swine Cartilage

Intact swine knees were collected as discarded tissue from other studies at Massachusetts General Hospital. Fresh articular cartilage was harvested as an osteochondral plug from the femoral condyle of knees from 3–4-month-old swine under sterile conditions using a 3 mm diameter biopsy punch. Holes of ~400 µm in diameter were then created via infrared laser ablation. For comparative purposes, similar diameter holes were created mechanically, using a coring needle (BD Insulin Syringe, 28G, Franklin Lakes, NJ, USA), to determine possible advantages or disadvantages of fractional laser treatment. The diameter and depth of the ablated channel in the osteochondral tissue of the articulating surface were controlled with the light delivery parameters (power, spot size, pulse duration, etc.) from a fractional 10.6 µm CO_2_ laser (UltraPulse^®^, Lumenis, Inc., San Jose, CA, USA). In both cases, lesions were placed at least 1 cm apart on the cartilage surface. In the case of the laser, a rastered delivery was used to create an array of lesions through multiple passes, the number of passes determining the depth of the ablated channels. Such fine depth control is not possible using mechanical coring needles, where all lesions are placed at an osteochondral depth.

### 2.6. Cartilage Generation Ex Vivo

**Figure 1:**
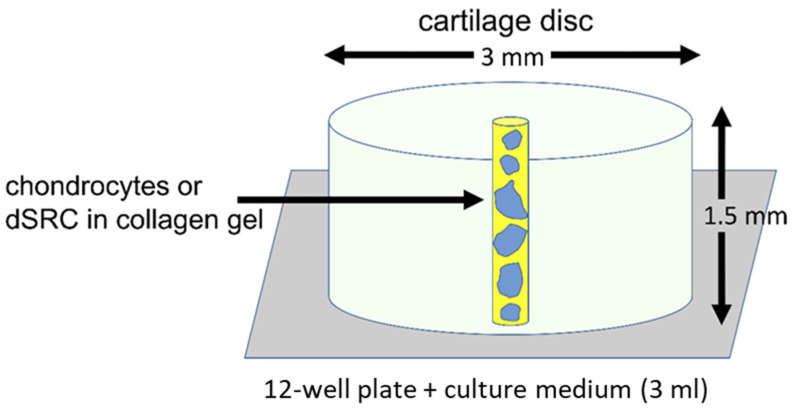
Schematic illustration of ex vivo experimental model.

After 14 days of culture in an oscillating motion, the dSRC aggregates were extracted from the culture, mixed with collagen gel (PureCol^®^ EZ Gel, Advanced BioMatrix, San Diego, CA, USA), and placed in a ~400 µm diameter lesion at the center of a 3 mm outer diameter swine articular cartilage disc (Figure 1). Four different groups were tested: (1) dSRC in a laser-ablated lesion; (2) dSRC in a mechanically punched hole; (3) cultured chondrocytes in a laser-ablated lesion; and (4) cultured chondrocytes in a mechanically punched hole. Groups 3 and 4 utilized chondrocytes that were cultured adherently in monolayer in tissue culture flasks (Falcon, Corning, NY, USA) for 14 days at 37 °C (one passage), then suspended in type I collagen gel (Advanced BioMatrix) at 3 × 10^7^ cells per mL and injected into the lesion. The latter groups act as ACI models. The cartilage discs were immediately placed in a medium (Ham’s F-12 media supplemented with 10% fetal bovine serum, 1% antibiotic/antimycotic solution, 1% nonessential amino acids (Invitrogen), and 50 mg/mL of ascorbic acid) at 37 °C.

### 2.7. Histological Analysis

dSRC samples (n ≥ 5) after multiple weeks of in vitro culture, ex vivo implant constructs from each group, and samples of native swine articular cartilage from the femoral condyle were fixed in 10% phosphate-buffered formalin for 48 h, embedded in paraffin, and then sectioned before staining with hematoxylin and eosin (H&E), Masson’s Trichrome, Safranin-O, and Toluidine Blue staining were also performed to assess chondrocyte density and the biochemical composition of the neomatrix. Whole slides were imaged using NanoZoomer 2.0HT Digital Pathology (Hamamatsu Photonics K.K., Hamamatsu, Japan) and analyzed using NDP.View2 software (Ver. 2.9, Hamamatsu).

### 2.8. Immunohistochemistry

Immunohistochemical staining for collagen types I and II was performed on tissue sections from each group (n ≥ 5). A rabbit anti-pig collagen I antibody (#AB138492, Abcam, Cambridge, MA, USA) and rabbit anti-pig collagen type II antibody (#AB34712, Abcam) were used. Antibodies were diluted 1:500 in 10% goat serum (Life Technologies, Thermo Fisher Scientific, Waltham, MA, USA) and 3% bovine serum albumin-blocking buffer (Sigma Aldrich) before use. Target Retrieval Solution (DAKO, Glostrup, Denmark #S1699, Ph 6.0–6.2) was used for antigen extraction. After 30 min in the blocking buffer, the slides were incubated with primary antibody for 60 min. In a negative control, no primary antibody was used. The slides were washed extensively in PBS (Fisher Scientific), incubated for 30 min with Mach 2 rabbit AP-Polymer secondary antibody (Dako, #K401011-2), and washed again in PBS (3 × 5 min). Immunostaining was developed using a Warp Red Chromogen kit (BioCare Medical Pacheco, CA, USA, #RALP525-G) for 15 min. Finally, the slides were washed in distilled water, and the nuclei were visualized with hematoxylin.

### 2.9. Live/Dead Assay

**Figure 2:**
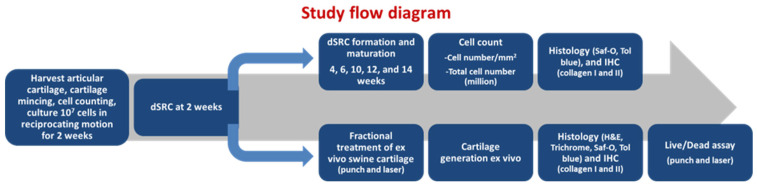
Flow diagram depicting all phases of the study.

Cell death caused by laser ablation or mechanical punch in freshly harvested swine articular cartilage discs was evaluated using Live/Dead fluorometric imaging. These discs were sharply divided into two pieces and cut through the center of the hole. The sections were then incubated with 150 µL of fluorescent dye solution (LIVE/DEAD^®^ Viability/Cytotoxicity Assay Kit; Molecular Probes, Eugene, OR, USA), consisting of 20 µL of calcein and 5 µL of ethidium homodimer in 10 mL of phosphate-buffered saline (PBS). Cell viability was assessed using a fluorescence microscope (Microphot-Fx; Nikon, Garden City, NY, USA). The calcein is taken up by viable cells and strongly fluoresces green; the ethidium homodimer passively enters nonviable cells and weakly fluoresces red. Figure 2 shows a flow diagram depicting all phases of the study.

### 2.10. Statistical Analysis

The data are presented as means ± standard deviation (SD). Statistical significance was determined using Student’s *t*-test with a statistically significant difference set at *p* < 0.05.

## 3. Results

### 3.1. Formation and Maturation of dSRC

**Figure 3:**
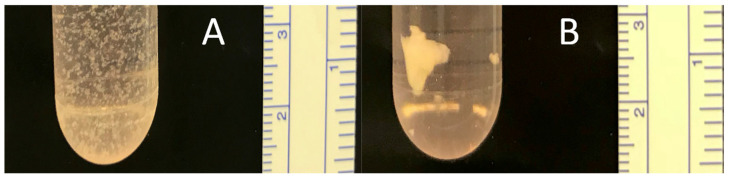
Photographs showing dSRC maturation after 2 (**A**) and 10 (**B**) weeks of dynamic reciprocating culture.

**Figure 4:**
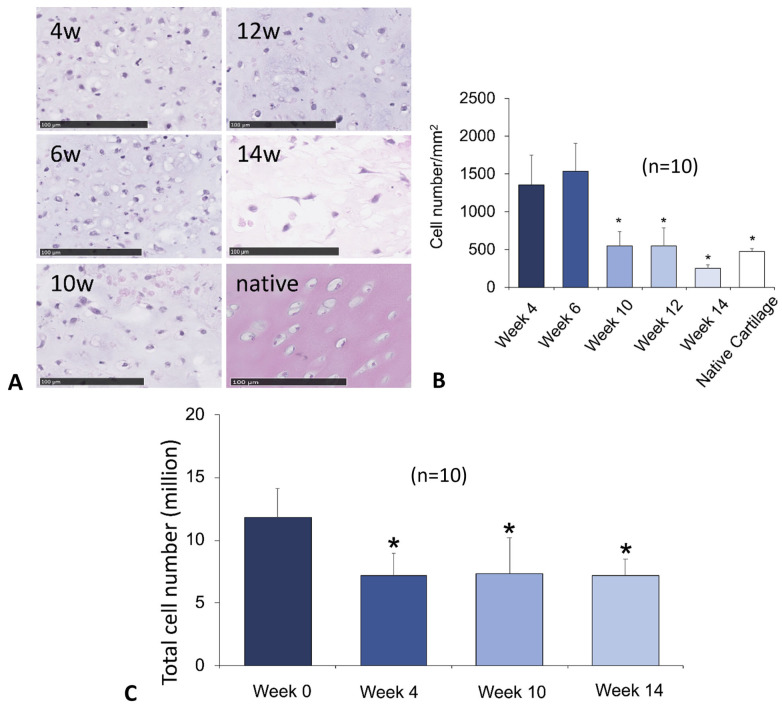
(**A**) H&E staining of dSRC cultured for up to 14 weeks in vitro. (Scale bars = 100 μm; insets 1 mm). (**B**) The graph shows a decrease in hypercellularity with dSRC maturation, eventually similar to native cartilage (n = 10). Asterisks denote a statistically significant difference (*p* < 0.05) compared to week 4. (**C**) Total cell number in dSRC samples over time up to 14 weeks, compared to the initial seeding number (n = 10). Asterisks denote a statistically significant difference (*p* < 0.05) compared to week 0.

**Figure 5:**
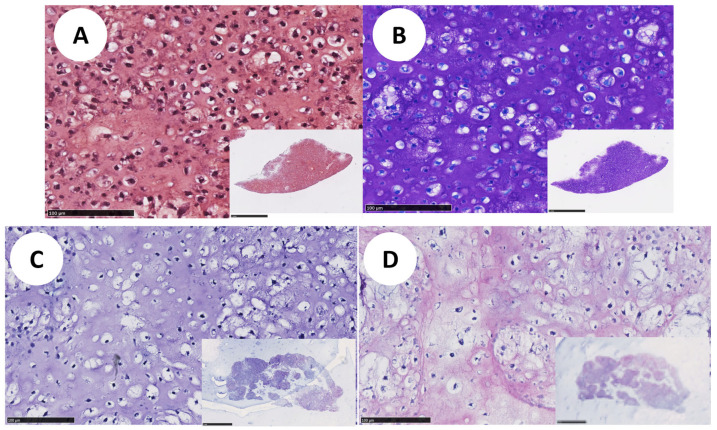
(**A**) Safranin-O and (**B**) Toluidine blue staining of dSRC matrix formation in culture after 10 weeks in vitro. Immunohistochemical staining for (**C**) collagen type I (**D**) and type II of dSRC after 10 weeks of culture in vitro (scale bars = 100 μm; insets 1 mm).

dSRC formation proceeds via initial chondrocyte microaggregation and ultimate coalescence into a larger, more solid pellet or sheet over weeks, as shown in Figure 3. Histologic sections show a contiguous neocartilage matrix after 8 weeks in vitro. A comparison of dSRC sections as a function of maturation time allowed for an assessment of relative cell density, as shown in Figure 4. One problem of encapsulated chondrocytes in hydrogel matrices is that a typical hypercellularity is not representative of true cartilage. Figure 4A,B show a significant decrease in cell density as dSRC matures and a decrease in the total cell count data in dSRC up to 14 weeks compared to the initial seeding number (Figure 4C). The total cell number did not change between week 4 and week 14, while the density decreased during this period (Figure 4C), in agreement with growth in matrix volume over time. The continual growth of the dSRC matrix, which was high in GAG content, was observed (Figure 5A,B). Immunohistochemical staining further demonstrated that the dSRC matrix appeared typical of hyaline cartilage, rich in collagen type II with no collagen type I, and similar to native cartilage (Figure 5C,D).

### 3.2. Fractional Treatment of Swine Articular Cartilage

**Figure 6:**
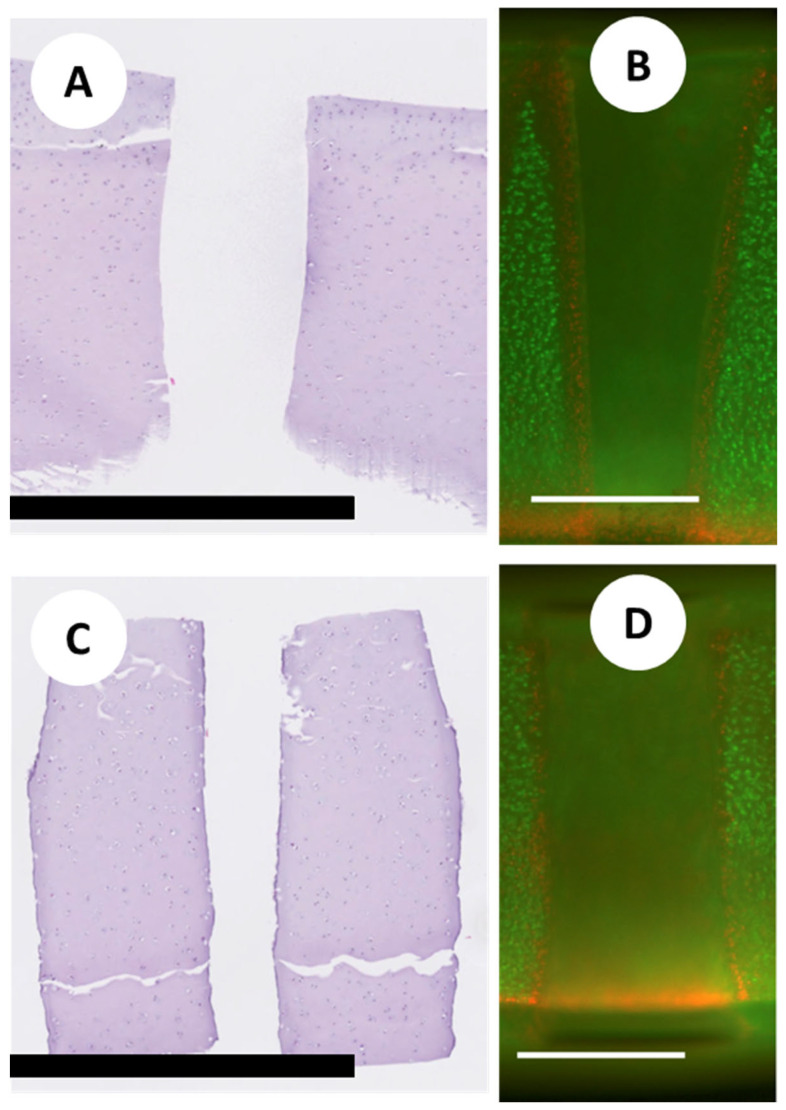
Photomicrographs of holes in cartilage discs created by a mechanical punch (**A**) or laser ablation (**C**) (scale bar = 1 mm). Live/Dead fluorescence micrographs showing live cells (green) in the native tissue outside the holes and dead cells (red) at the interface of the hole and native tissue for laser-ablated (**B**) and mechanically punched holes (**D**) (scale bars = 500 μm).

Laser ablation produced lesions of varying depth and diameter depending on the dosimetry parameters. Both laser ablative and mechanical approaches produced lesions of ~400 µm in diameter, extending to depths of 1–1.5 mm). Figure 6A,B show comparative micrographs of laser-ablated and coring needle lesions. The photothermal effect of laser ablation on the native cartilage tissue was investigated using Live/Dead fluorescence microscopy. Figure 6C,D also show fluorescence micrographs highlighting the shape of the channels and the loss of viability at the channel interface with native cartilage. Under the conditions used, the mechanically punched holes have a slightly greater diameter than laser-ablated channels, and the diameter is relatively constant with channel depth. Laser-ablated channels are conical due to the laser beam optics, with the diameter narrowing with depth. Both punch and laser are similar regarding surface smoothness. In both cases, there is a thin (<50 µm) superficial layer of loss of viability in the channels, which perhaps extends slightly deeper for the laser-ablated channels.

**Figure 7:**
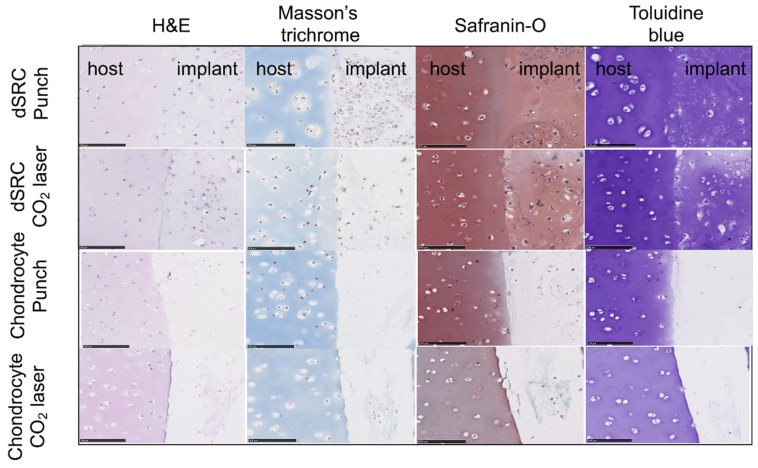
H&E, Masson’s trichrome, Safranin-O, and Toluidine blue staining of cartilage matrix after 8 weeks in mechanical and laser-ablated defects in vitro (scale bars = 100 μm).

**Figure 8:**
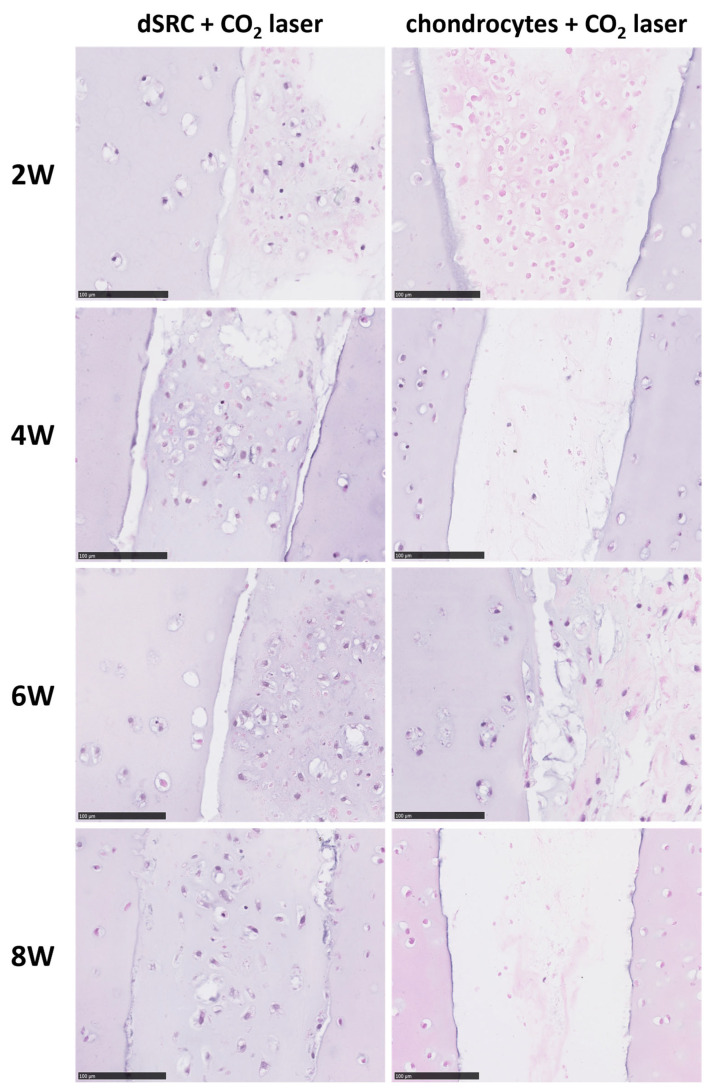
H&E staining of cartilage matrix after 2, 4, 6, and 8 weeks in laser-ablated defects filled with dSRC and chondrocytes in vitro (scale bars = 100 μm).

**Figure 9:**
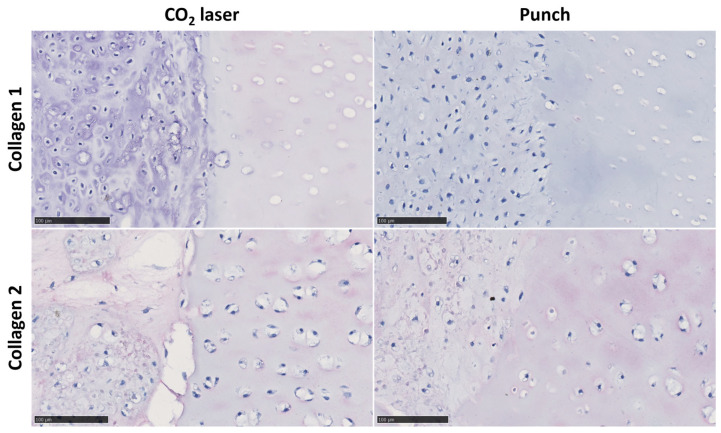
Immunohistochemical staining for collagen type I and type II of dSRC in mechanical and laser-ablated defects after 10 weeks of culture in vitro (scale bars = 100 μm).

At week 8 after implantation, the matrix formed by the dSRC group in both laser-ablated and mechanically punched holes demonstrated noticeable improvement over encapsulated chondrocytes in terms of contiguous matrix and composition (Figure 7). Figure 7 also shows clearly that the neocartilage matrix produced via dSRC is well integrated at the native cartilage interface. In contrast, the encapsulated chondrocyte preparation does not integrate well. The dSRC groups generated more GAG than isolated chondrocytes. The maturation of the neotissue is shown for both implant types as a function of time in Figure 8. Results show less consistent neotissue generation with a lack of contiguity for the chondrocyte samples, while the dSRC samples show the gradual maturation and integration of neocartilage with the host, as underlined at 8 weeks in Figure 7. Immunohistochemical staining showed that the matrix made by dSRC groups in either lesion stained positively for type II collagen but not for type I collagen, further indicating that the matrix is characteristic of hyaline cartilage (Figure 9).

## 4. Discussion

An unmet need in orthopedics is a new restorative method for treating cartilage lesions in the knee joint. We previously reported that dSRC promotes contiguous hyaline articular cartilage generation in a rodent model of focal lesions in vivo and that hydrogel-capped dSRC generated an articular cartilage matrix in swine in non-load-bearing environments [25]. The size of the cartilage defect in the prior swine study was much larger than here, with a 2 mm diameter and 5 mm depth lesion created by a mechanical punch.

This study evaluated the maturation of the dSRC matrix, cultured under reciprocating movement, at different time points. This information is important for two reasons: (1) to determine how dSRC matures over extended times in culture and (2) to determine the appropriate time before transplantation into fractional defects. On a gross level, the dSRC particles became larger and more solid with time in culture. dSRC successfully generates a contiguous articular cartilage matrix in a suitable in vitro environment. After 2 weeks, the small dSRC particles remain injectable through a 28 g needle. By 12 weeks, the larger and more mature dSRC can be manually placed in larger lesions (>2 mm in diameter). As the dSRC matures in culture, the hypercellularity of the matrix gradually decreases, the dSRC becomes stiffer, and the nodule size increases. Histology and immunostaining further confirmed that the 14-week dSRC matrix was characteristic of hyaline cartilage, with a high GAG content, rich in collagen type II, and lacking collagen type I. Implanting such mature dSRC would not be ideal, as dSRC maturation should occur under physiological stress in vivo and in situ. The immature dSRC after two weeks of in vitro culture under reciprocating motion is ideal for implantation and further maturation in situ. The reduced time between chondrocyte harvest and implantation is advantageous for clinical application, avoiding the lengthy culture, expansion, and potential loss of chondrogenic phenotype. There are also other potential advantages, as it may be possible to use alternative and less invasive articular chondrocyte sources (e.g., auricular).

One advantage of resurfacing through sub-mm-diameter fractional treatment is the potential regeneration of normal cartilage tissue without scarring. This is not the subject of this study, as it requires an in vivo study, but we can evaluate the potential suitability of the dSRC implant approach in such lesions. Sub-mm-diameter microlesions were created using a mechanical (coring needle) or a photoablation (fractional CO_2_ laser) approach. The mechanical method is simple but tedious, whereas the laser method is rapid but costly, with the added advantage that parameters (diameter, depth) can be tightly controlled. Laser ablation will always be accompanied by a degree of collateral thermal damage to tissue. Still, in this comparative study, there is a similar in cell viability at the channel interface following mechanical punch and laser ablation, with only very superficial effects observed in each case. Additionally, there were no noticeable differences in the matrix formed via dSRC in channels created mechanically or through laser ablation. Thus, any acute photothermal damage produced at the channel interface has no adverse effect on long-term cartilage formation or as a barrier to integrating the neomatrix with native cartilage.

The advantages that may be presented through laser ablation treatment in joint disease include the exquisite control of prepping the tissue to receive implants and avoiding sequelae related to bleeding that could interfere with implant placement. This study suggests that the functionality and integration of dSRC have great potential in regenerative treatment in the joints. Considering all the above, the fractional laser approach is appealing for further in vivo studies to facilitate cartilage regeneration and integration.

We previously demonstrated that dSRC can generate an excellent hyaline cartilage matrix in vitro in large focal cartilage lesions. In the current application, the dSRC placed into punch- or laser-drilled microlesions in swine articular cartilage discs ex vivo produces a very similar matrix to native cartilage and integrates well with the native cartilage. This may be suggestive of the biomechanical stability of the overall host-neocartilage tissue. Interestingly, the migration of the chondrocytes from the dSRC into the native cartilage surface was observed ex vivo with H&E staining (Figure 8). Safranin-O and Toluidine blue staining further confirmed that the dSRC matrix reflects the hyaline cartilage matrix, demonstrating that dSRC produced significantly more extracellular matrix components than the encapsulated chondrocyte group. Immunohistochemical staining also supports the idea that the matrix reflects hyaline cartilage rather than fibrocartilage. Although the early phase of growth of dSRC in vitro has some limitations, such as initial hypercellularity and non-uniform composition, the dSRC matrix is still similar to the native cartilage matrix. One would expect the further maturation of the neocartilage to occur with time. The rapid integration with host tissue is a major advantage of this approach in comparison to others, ultimately generating truly functional articular cartilage tissue, especially under physiological stimulation in vivo.

In our previous study [25], a mechanical punch was used to create lesions. These were inevitably of an osteochondral nature, and penetration into the bone marrow at the osteochondral junction causes bleeding. In an ongoing in vivo study that is outside the scope of this work, we have utilized laser ablation as a means for the exquisite control of lesion depth, with the ability to confine lesions within the cartilage layer. The mechanistic study reported here allowed us to test whether the laser ablation mechanism would have any detrimental effects on the host tissue (e.g., thermal damage to tissue and cytotoxicity) or affect the host/implant interface that could negatively impact the integration of neotissue with the host. The fact that we do not observe any detrimental effects of laser ablation opens up its use in the preparation and possibly pre-sculpting of host lesions to receive dSRC implants. The smaller size of the holes prepared in this study compared to the Meppelink et al. study allowed us to focus more on the interface with multiple lesions created per sample while still comparing the mechanical vs. laser preparation of lesions [25]. We might also add that we are currently considering treatment approaches for resurfacing superficial cartilage disease with the potential for fractional laser rejuvenation, with and without dSRC. This study also serves as a precursor for that work in terms of how the dSRC would behave in the microscopic lesions created by fractional infrared laser systems, similar to those used for skin rejuvenation [27,28].

Although promising, these studies have distinct limitations. Our analyses of dSRC implantation were all performed ex vivo, thus lacking the tissue’s in situ biological responses due to the creation of the fractional microlesions. One complicating factor in our previous studies of osteochondral defects was intraoperative bleeding from the bone marrow that welled into the focal lesion, which complicated the placement of encapsulated chondrocyte hydrogels [25]. Laser ablation solves this problem via controlled dosimetry containing the lesion to the cartilage layer and a photothermal coagulative effect accompanying photoablation [29,30,31,32]. Additionally, the defects were not subject to forces equivalent to normal weight bearing, and in vivo experiments are required to investigate such influences. Finally, as with other ACI techniques, a source of autologous donor chondrocytes is required, necessitating harvest surgery. This may not require articular cartilage, as previous studies have demonstrated good cartilage generation using costal or auricular chondrocytes [33,34]. Nevertheless, without immunosuppression, an autologous source would be required for dSRC production. Tightly controlled fractional treatment lasers are widely commercially available for skin rejuvenation indications and require minimal modification for cartilage resurfacing but are nonetheless expensive.

## 5. Conclusions

The dSRC maturation process produces neocartilage that becomes more typical of hyaline cartilage formation with time. The combination of fractional laser treatment and dSRC successfully produces an integrated hyaline matrix in microlesions in native cartilage ex vivo. Future testing of this combinatorial approach for cartilage regeneration and resurfacing will focus on the longer-term effects of fractional treatment in a swine articular cartilage (knee) model where in vivo mechanical forces on the regenerating matrix are recapitulated.

## Data Availability

The data presented in this study are available upon reasonable request from the corresponding author.
